# Evidence for Disruption of Mg^2+^ Pair as a Resistance Mechanism Against HIV-1 Integrase Strand Transfer Inhibitors

**DOI:** 10.3389/fmolb.2020.00170

**Published:** 2020-08-20

**Authors:** Lucas de Almeida Machado, Ana Carolina Ramos Guimarães

**Affiliations:** Laboratory for Functional Genomics and Bioinformatics, Instituto Oswaldo Cruz, Fiocruz, Rio de Janeiro, Brazil

**Keywords:** HIV-1, integrase, intasome, resistance, molecular dynamics

## Abstract

HIV-1 integrase is the enzyme responsible for integrating the viral DNA into the host genome and is one of the main targets for antiretroviral therapy; however, there are documented cases of resistance against all the currently used integrase strand transfer inhibitors (INSTIs). While some resistance-related mutations occur near the inhibitor’s binding site, the mutation N155H occurs on the opposite side of the drug-interacting Mg^2+^ ions, thus, not interacting directly with the drug molecules and currently lacking an explanation for its resistance mechanism. Moreover, mutation N155H and the resistance-related mutation Q148H are mutually exclusive for unknown reasons. In the present study, we use molecular dynamics simulations to understand the impact of the N155H mutation in the HIV-1 integrase structure and dynamics, when alone or in combination with Q148H. Our findings suggest that the Mg^2+^ ions of the active site adopt different orientations in each of the mutants, causing the catalytic triad residues involved in the ion coordination to adapt their side-chain configurations, completely changing the INSTIs binding site. The change in the ion coordination also seems to affect the flexibility of the terminal viral DNA nucleotide near the active site, potentially impairing the induced-fit mechanism of the drugs. The explanations obtained from our simulations corroborate previous hypotheses drawn from crystallographic studies. The proposed resistance mechanism can also explain the resistance caused by other mutations that take place in the same region of the integrase and help uncover the structural details of other HIV-1 resistance mechanisms.

## Introduction

The current guidelines for AIDS treatment rely mainly on inhibitors of the viral reverse transcriptase and integrase (IN) (Cahn et al., 2019a,b). The human immunodeficiency virus type 1 (HIV-1) IN is a 288 residue protein responsible for integrating the viral DNA (vDNA) into the host genome (Blanco et al., 2011). The IN structure comprises an N-terminal domain (NTD) and a C-terminal domain (CTD) mainly responsible for DNA binding and enzyme multimerization, and a catalytic core domain (CCD) (Hare et al., 2010a). The enzyme accomplishes the integration process by carrying out two reactions: (i) cleavage of two or three nucleotides at the 3’ end of the viral DNA and (ii) the strand transfer reaction, where the vDNA is covalently bonded to the host DNA (tDNA) (Craigie, 2001; Blanco et al., 2011). Given the central role of this process in the viral replication process, the use of IN as a target of antiretroviral therapy is intuitive.

To date, the strand transfer inhibitors (INSTIs) are the only class of IN inhibitors used in therapy (Arts and Hazuda, 2012). The INSTIs are divided into two generations: the first, which includes Raltegravir (RAL) and Elvitegravir (EVG); and the second, represented by Dolutegravir (DTG) and more recently Bictegravir (BIC) (Fesen et al., 1993; Evering and Markowitz, 2007; Shimura and Kodama, 2009; Blanco et al., 2011; Pendri et al., 2011; Akil et al., 2015; Tsiang et al., 2016). The INSTIs bind to the vDNA-enzyme complex after the 3′ cleavage of the vDNA, in the so-called cleaved stable synaptic complex (cSSC) (Li and Craigie, 2009), before the formation of the complex between vDNA and tDNA and the strand transfer reaction. One important thing in common among the currently used inhibitors is the diketoacidic scaffolds that can chelate divalent cations and bind the Mg^2+^ ion pair at the active site (Hare et al., 2010b; DeAnda et al., 2013).

Despite the effectiveness of the current IN inhibitors, many HIV-1 resistance-related mutations are known to emerge in patients treated with INSTIs (Charpentier et al., 2008; Cooper et al., 2008; Miller et al., 2008; Malet et al., 2009). There are three commonly frequent resistance pathways among the resistance mutations: N155H, Q148HRK, and Y143HC; interestingly, N155H and Q148HRK are mutually exclusive (Fransen et al., 2008; Miller et al., 2008; Malet et al., 2009). A previous study using the structure of the CCD of the HIV-1 IN predicted that some potential inhibitors (including RAL) make contact with residue N155, among others (Serrao et al., 2009). However, the study was carried out using only the structure of IN CCD. Careful inspection of the prototype foamy virus (PFV) in complex with RAL (Hare et al., 2010b) shows that residue N224—which is equivalent to the N155 in HIV-1 integrase—does not directly interact with the inhibitors and is located on the opposite side of the two drug-interacting Mg^2+^ ions. This observation raises questions about the resistance mechanism behind the N155H variant since the mutated residue does not interact directly with the drug molecules.

Grobler et al. (2008) suggested that the mutation N155H causes resistance by perturbing the arrangement of Mg^2+^ in the active site. Moreover, experiments with the N224H mutant of the PFV showed that the mutant only binds one ion in the active site (Hare et al., 2010a). The recent determination of the HIV-1 IN STC structure by cryo-EM (Passos et al., 2017) is a breakthrough that allows a detailed analysis of the architecture of the enzyme in complex with its substrate. Here, we use information from the cryo-EM STC structure of the HIV-1 IN to build models of cSSC of the wild-type (WT) IN, the N155H variant, and the double mutant N155H+Q148H. These models were used for molecular dynamics (MD) simulations to investigate the effects of the mutations on the structure and dynamics of the main actors on the active site. The simulations were used to better understand the resistance mechanisms of N155H, as well as the reasons why N155H and Q148H are mutually exclusive. Here, we also investigate the overall dynamics of the cSSC.

## Materials and Methods

### Comparative Modeling

The consensus sequence of the HIV-1 integrase of subtype B (UniProt accession code: B9VIC1) was used as reference to build the structure of the cSSC of each system (WT, N155H, and N155H+Q148H) through comparative modeling. One of the templates used was the HIV-1 strand transfer complex (STC) structure obtained by cryo-EM (PDB code: 5u1c) (Shimura and Kodama, 2009). This structure is a tetramer of IN around the vDNA after the integration to the tDNA; it is comprised of two inner chains (A and C) in contact with the vDNA and two outer chains (B and D) which interact, respectively, with A and C. Therefore, we removed the tDNA segment to model the cSSC, i.e., the complex before the strand transfer reaction. The cryo-EM structure lacks coordinates for residues 205–222 of the inner chains and residues 187–217 from the outer chains. To model these segments, we used a crystal structure of the HIV-1 integrase (1ex4) (Van Der Spoel et al., 2005). Another information lacking in the cryo-EM structure is the coordinates of the most internal Mg^2+^ ions of the active site. To model a complex with both Mg^2+^ atoms, we used the coordinates of the Mg^2+^ ions of the PFV intasome structure (3OYA) (Arts and Hazuda, 2012), given the conservation of the active site structure between both enzymes (Kulkosky et al., 1992; Hare et al., 2010a). Here, we named the Mg^2+^ ion closer to the surface as Mgα and the most internal and closer to residue 155 as Mgβ. Twenty models were generated with Modeller 9.18 (Fiser and Šali, 2003). The model with the lowest DOPE score of each system was validated by inspection of its stereochemical properties using the PROCHECK server (Fiser and Šali, 2003) and then used for the MD simulations. The final models had two inner chains (A and C) ranging from residue 1 to 269 and two outer chains (B and D), ranging from residue 58 to 269. Hydrogens were added to the modeled structures according to the most probable protonation state of the titratable residues in each system at pH 7.0, calculated using the software propka (Olsson et al., 2011).

### Molecular Dynamics

The systems were solvated in dodecahedral boxes of water, using the TIP3P water model. The size of the boxes was estimated to assure that the protein or DNA was 10 Å distant from the edges. Afterward, the systems were neutralized by the addition of enough Na^+^ ions. In the next step, all systems went through an energy minimization with the steepest descent algorithm, using a maximum of 100000 steps and a maximum force of 0.01 KJ mol^–1^ nm^–1^ as convergence criterion. After the minimization step, each of the systems was divided into three replicas. For the heating step, each of the replicas was assigned velocities using different random seeds for atom velocity generation and then heated to 310 K during the first 0.5 ns of a 1 ns MD run in an NVT ensemble. Temperature coupling was accomplished using the v-rescale thermostat (Bussi et al., 2007), and the heating was carried out using position restraints. The heating step of each replica was followed by a 1.5 ns NPT run maintaining the position restraints, and another 1.5 ns NPT run without the position restraints, both using the Berendsen barostat for pressure coupling (Berendsen et al., 1984). Then, 100 ns production simulations were carried out for the three replicas of each system, using the v-rescale thermostat and Parrinello-Rahman barostat (Parrinello and Rahman, 1981). All steps were done using the Gromacs 2018 software (Van Der Spoel et al., 2005) and the forcefield Amber-ff99sb-ildn (Lindorff-Larsen et al., 2010). Bonds between hydrogen and heavy atoms were constrained. A time step of 2 fs was used and frames were written every 20 ps. Electrostatic interactions were handled using Particle Mesh Ewald, Fourier spacing was set to 0.12 nm and PME order to 4. The Ewald-shifted direct potential at the cutoff was 1 × 10^–5^ (Darden et al., 1993; Essmann et al., 1995). For Van der Waals interactions, a cutoff of 1 nm was used.

The root-mean-square deviation (RMSD) was calculated for each frame of the MD simulations in relation to the starting structures. The snapshots generated after the RMSD of each replica reached equilibrium were compiled for each system and used to calculate the root-mean-square fluctuations (RMSF) and to analyze the non-bonded interactions. The RMSD equilibrium was defined as a contiguous set of frames within a replica where the correlation between time and RMSD is between -0.35 and 0.35. The compiled snapshots were also used to generate a set of clusters based on the RMSD of the protein backbone and another set of clusters using the vDNA RMSD, both using a 2 Å RMSD cutoff. The distances between N1 of the cytosine and N9 of the terminal adenine were calculated through the whole simulations in chain A and chain C, in order to investigate the dynamics of the terminal nucleotide near the active site.

## Results

### Comparative Modeling

**FIGURE 1:**
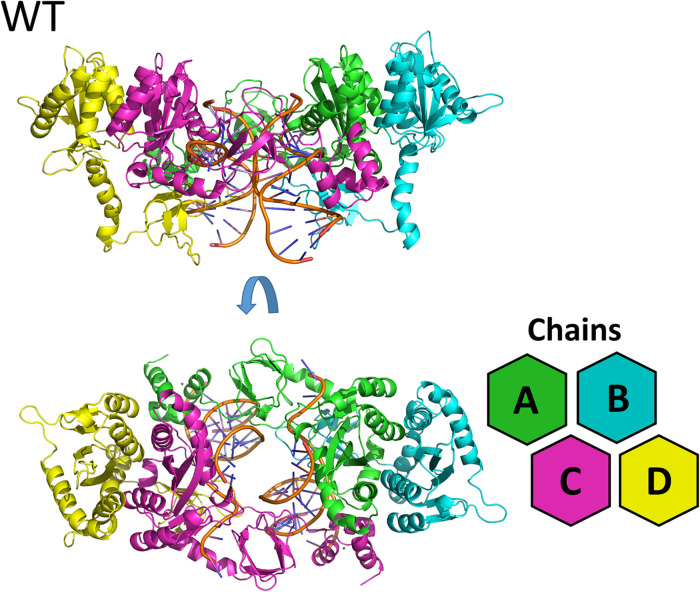
WT cSSC model. Top and front view of the cSSC complex of the WT protein detail the spatial organization of the IN monomers. The inner chains (A and C) and outer chains (B and D) are identified by color; the vDNA strands are located in the center of the complex, colored in orange.

The lowest dope score models constructed for the cSSC of WT, N155H, and N155H+Q148H showed ≈98% of the residues in the allowed and favored regions of the Ramachandran plot. Analysis with PROCHECK server also showed that the stereochemical properties of the model are within the expected values for a structure of ≈1.5 Å resolution, except for slight deviations of the peptide bond planarity and G-factor. In the models, the catalytic triad and the terminal adenine were coordinating the Mg^2+^ ion pairs, according to its known spatial configuration (Hare et al., 2010b; Passos et al., 2017). The RMSD between both mutants and the WT intasome was ≈1.6 Å, showing that the point mutations caused no major structural changes, and the differences occur mostly in loop regions. It is possible to identify in the structure both inner chains (A and C)—close to the vDNA—and both outer chains (B and D), which do not participate directly in the catalysis (Figure 1). The three complexes can be seen in Supplementary Figure S1. We also compared our WT structure with two recently published structures that were not available by the time of the study: the SIV intasome recently published by Cook et al. (2020) (PDB code 6RWN) and the cleaved synaptic complex structure published by Passos et al. (2020) (PDB code 6V3K). The comparison of the inner chains showed an RMSD of 1.2 Å and 1.3 Å when compared with 6RWN and 6V3K, respectively. Moreover, both comparisons showed an almost perfect superposition of the Mg^2+^ ions at the active site (Supplementary Figure S2). Regarding the protonation, all H155 and H148 were predicted as δ-protonated.

### Overall Dynamics of the Complexes

**FIGURE 2:**
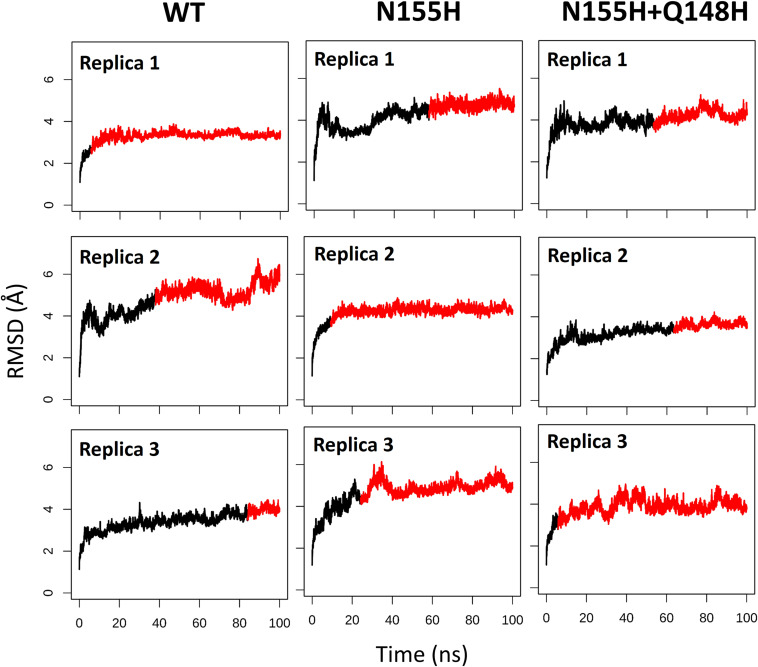
RMSD of each replica of the WT, N155H, and N155H+Q148H systems. The graphs show the RMSD of the protein backbone as a function of time for each of the three replicas of each system. The red region of the lines corresponds to the simulation from the point where the backbone RMSD reached equilibrium.

**FIGURE 3:**
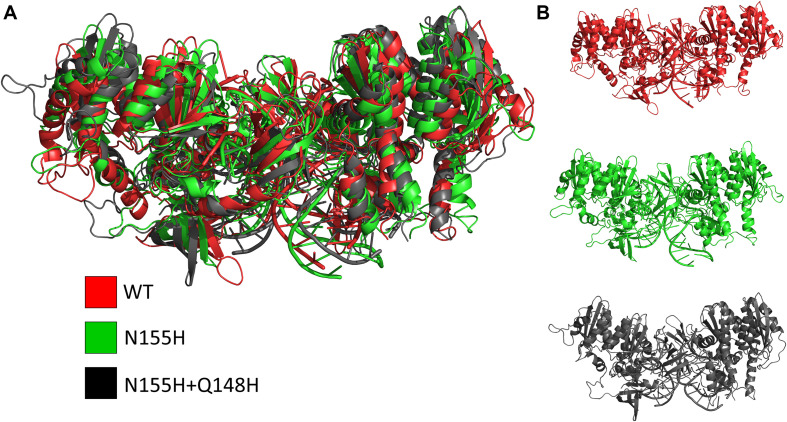
Cluster centroids of the WT, N155H, and N155H+Q148H complexes. The superposition of the cluster centroids from all the systems is shown in **(A)**; WT, N155H, and N155H+Q148H are shown, respectively, in red, green, and black; panel **(B)** shows the cluster centroids individually.

**FIGURE 4:**
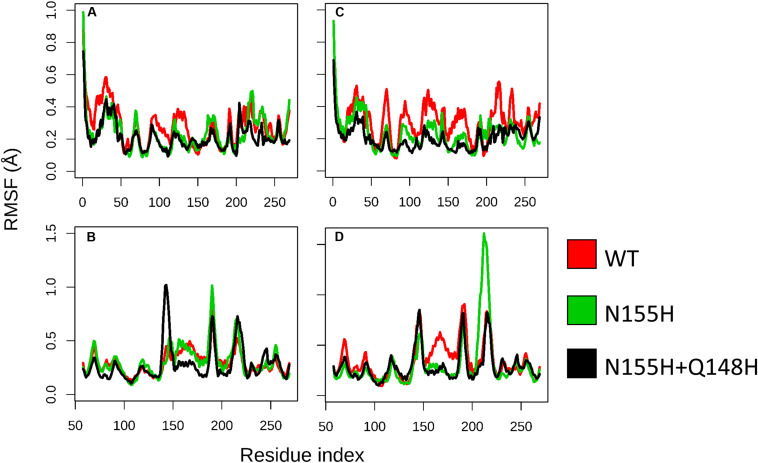
RMSF of WT, N155H, and N155H+Q148H. Each graph displays the RMSF of one of the cSSC chains **(A–D**) calculated from the MD replicas of each system after reaching equilibrium. RMSF of WT is shown in red, N155H in green, and the double mutant in black. The patterns of fluctuation are different in the inner chains **(A,C)** when compared to the outer chains **(B,D)**, given the steric hindrance caused by the contact of the inner chains with the DNA, with the outer chains, and with each other.

Except for replica 1 of the WT intasome, replica 1 of the N155H variant, and replicas 1 and 2 of the double mutant, all simulations reached equilibrium before 50 ns (Figure 2). The centroids of the clusters obtained from the MD trajectories of the systems showed slightly different configurations of the Mg^2+^ ions of the active site when compared to the WT, as seen in Figure 3. These configurations are consistent throughout all the cluster centroids and were observed in all the replicas. The so-called flexible loop (residues 140–149), regarded by many studies as a flexible part that plays a role in catalysis (Barreca et al., 2003; Williams and Essex, 2009), showed higher fluctuations in the WT, which was even more pronounced in chain C, as seen in Figure 4. On the other hand, the region from residue 100–150 is much less mobile in both mutants, which is one possible explanation for the catalytic deficiency of the N155H (Marinello et al., 2008); moreover, in chain C, the region around residue 155 is also less motile than the WT. The RMSF patterns in the outer chains interestingly resemble the RMSF observed by Williams and Essex (2009) and the B-factors calculated from simulations by Barreca et al. (2003), when the authors exclusively simulated the dynamics of the CCD domain of the HIV-1 IN. Both studies observed fluctuation peaks on the so-called flexible loop and in a region close to residue 180, and both patterns visually resemble the RMSF plots of our complexes’ outer chains. Besides using a single domain, the structures used in the aforementioned studies lack one Mg^2+^ atom and DNA chains near the active site, which is a condition similar to that of the outer chains of the cSSC, and probably explains the increased flexibility observed in these previous studies. The presence of mutation Q148H seems to cause a peak in chain B exactly in the vicinity of the mutated residue.

### Mg^2+^ Ions Coordination

**FIGURE 5:**
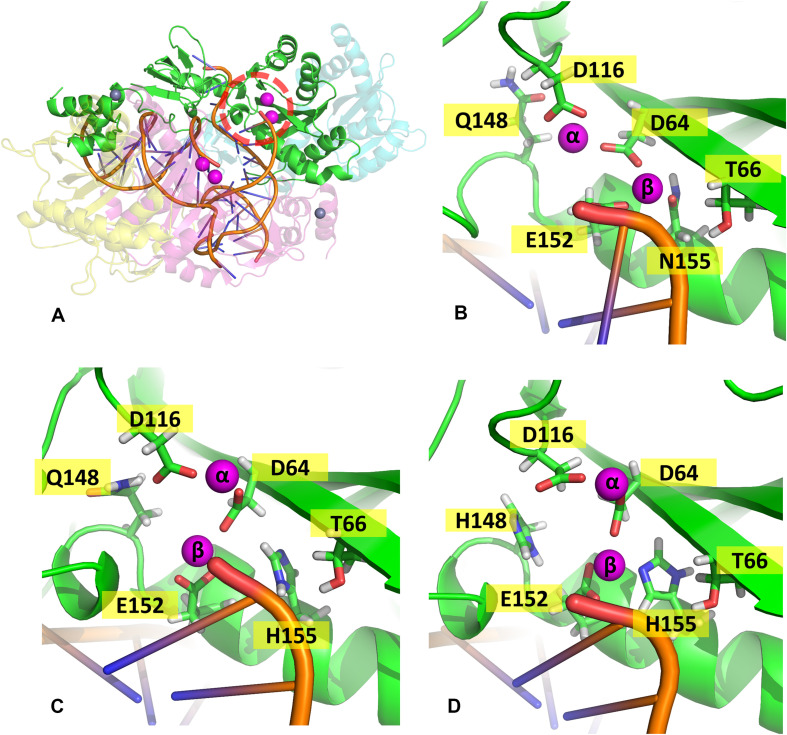
Coordination site of the Mg^2+^ ions. Panel **(A)** shows the whole complex, and the red dotted circle indicates the localization of chain **(A)** active site; panels **(B–D)** show the coordination site of the Mg^2+^ ions in chain A from one of the cluster centroids of WT, N155H, and N155H+Q148H, respectively. The Mg^2+^ ions are shown in magenta and the DNA backbone in orange.

In the mutant proteins, the coordination of the Mg^2+^ ions by the catalytic triad (D64, D116, and E152) is different from the WT enzyme (Figure 5). In both mutants, the side chain of D64 is rotated to accommodate the new position of the ions, while the loop containing D116 is displaced, so the coordination of Mgα is maintained, while E152 is also slightly rotated. Even the terminal adenine of the vDNA changes how it coordinates the ions in both variants, only contacting Mgβ. The position of the ions is tilted in relation to the WT coordinates in both mutants; however, in the double mutant, Mgβ is shown closer to residue 155, while in N155H, the Mgβ is coordinated much further from H155. These pieces of evidence reinforce the hypothesis that the mutation cause resistance by altering the position of the ions (Grobler et al., 2008). Since the INSTIs metal-chelating group interacts with the ion pair (Hare et al., 2010b), it is reasonable to infer that the INSTIs would bind in a completely different pose in the mutant, if binding at all. The change in the active site could also explain the catalytic inefficiency of the mutant enzyme (Dicker et al., 2008), and in the case of the double mutant, it could explain why this combination of mutations is not positively selected *in vivo* (Charpentier et al., 2008).

**FIGURE 6:**
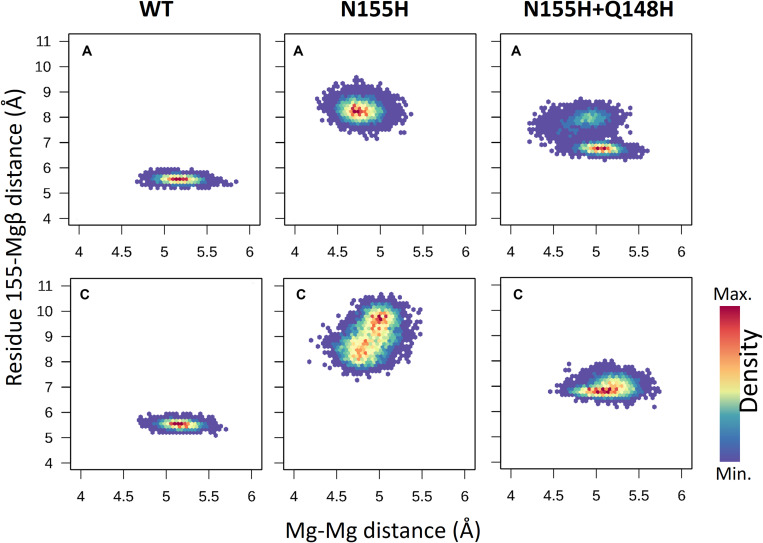
Mg–Mg and N/H–Mgβ distances. The graphs depict the Mg–Mg distances explored throughout the MD simulations in each system in relation to the 155-Mgβ distance. The color scale shows the relative density of frames that visited a given state.

When it comes to the dynamics of the ions, the distance between the two Mg^2+^ atoms increased in ≈0.5 Å on average in the N155H variant (Figure 6). In this mutant, the distance from the alpha carbon of residue 155 to Mgβ is also increased by ≈6 Å on average when looking at chain C. The double mutant shows a similar Mg–Mg distance as the WT and explores slightly higher distances between residue 155 to Mgβ, and in chain A, it displays two distinct populations of coordination states. Figure 6 also shows that while the WT enzyme displays a narrow window of distances between N155 and Mgβ, the mutants explore a wider variety of distances in both chains.

In the WT IN, Mgβ is in contact with T66, while in both variants, a new contact is formed with Q148, as the ion is displaced toward the so-called flexible loop, and the presence of histidine in position 148 further influences the coordination of Mgβ. It is important to note that T66 is a residue also involved in resistance events when mutated to isoleucine or lysine (Charpentier et al., 2008; Shimura et al., 2008; Gatell et al., 2010; McColl and Chen, 2010; Hurt et al., 2013). We believe that the resistance mechanisms of T66K and T66I could occur for similar events of ion displacement. The T66I mutant could displace the ion through the introduction of an apolar and longer chain, and the T66K mutant could cause ion displacement through the introduction of a positively charged longer chain close to the divalent cation. This hypothesis is also supported by the fact that N155H and T66I have similar EC50 profiles (Dicker et al., 2008).

The interaction energy between residue 155 and the Mgβ is lost in the single mutant; going from –222 KJ/mol (±11.9) to 1.2 KJ/mol (±4.1) (Table 1), these observations are consistent with the displacement of the ions further away from residue 155. Moreover, the adaptation of the E152 side chain to maintain the interaction with the Mgβ changed the interaction energy of the pair from -404.7 KJ/mol (±13.9) in the WT to -623.3 KJ/mol (±17.9) in the N155H mutant. D116 also showed more favorable interaction energy with the Mgβ ion in the N155H variant (≈35 KJ/mol difference), while D64 showed no significant difference. Additionally, the interactions between the Mg atoms in both mutants also become more repulsive due to the increased proximity between the ion pair. The double mutant displays huge differences in the interaction energy between D116 and Mgα (≈200 KJ/mol difference) when compared with WT and N155H; apart from this, all other catalytic residues interact with Mgα with very similar energy values in all systems. In the double mutant, the interaction between Mgβ and residue 155 is also practically lost, while the interaction energy with E152 is increased by ≈200 KJ/mol; but it did not show interactions between Mgβ and D116 as favorable as the N155H mutant. Importantly, the substitution in position 148 to histidine causes a more favorable interaction between residue 148 and Mgβ, but the high standard deviation suggests high fluctuations in the interaction. Another major difference in the coordination of Mgα in the mutants is the increased distance from the terminal adenine of the vDNA end, which does not coordinate the ion of the mutant enzymes.

**TABLE 1:** Interaction energies between Mgβ, Mgα, the terminal adenine, and catalytic and mutated residues in the cSSC complexes.

					**N155H+**	
	**WT**		**N155**		**Q148H**	
	**Energy**		**Energy**		**Energy**	
	**(KJ/mol)**		**(KJ/mol)**		**(KJ/mol)**	
**Pair**	**Mean**	**SD**	**Mean**	**SD**	**Mean**	**SD**
Mgα-Mgβ	24.3	4.8	43.7	7.1	35.3	8.3
D64-Mgα	–383.3	9.6	–380.2	12.2	–385.3	13.1
D116-Mgα	–590.3	19.0	–592.6	19.2	–379.3	11.0
Q/H148-Mgα	–23.5	11.7	–1.9	0.9	–2.0	1.0
E152-Mgα	–33.3	10.5	–3.2	0.6	–2.9	0.7
N/H155-Mgα	–0.7	0.2	0.1	0.2	0.0	0.0
D64-Mgβ	–388.0	11.5	–387.9	10.3	–385.0	10.5
D116-Mgβ	–3.3	1.3	–38.5	9.0	–10.0	8.5
Q/H148-Mgβ	–0.3	0.1	–14.6	4.1	–49.2	65.7
E152-Mgβ	–404.7	13.9	–623.3	17.9	–618.5	18.7
155-Mgβ	–222.0	11.9	1.2	4.1	2.0	1.4
DA-Q/H148	–1.0	0.8	–0.7	0.2	–0.9	0.3
DA-N/H155	18.4	6.3	–13.0	4.7	–15.8	11.1
DA-Mgα	0.8	2.8	–0.7	0.7	1.4	2.4
DA-Mgβ	–316.5	13.7	–147.2	16.4	–123.0	62.9

*The table depicts the interaction energies between the main actors in the active site in chain A. It shows the interaction energy averaged through the frames of the compiled MD simulations and the standard deviations in each system.*

In a study with crystals of the PFV IN soaked with INSTIs in a mutant equivalent to N155H, Hare et al. (2010b), the crystals made from mutant proteins soaked with inhibitors had only one Mn^2+^ (used instead of Mg^2+^ for crystallography purposes) in the active site. Possibly, the altered position of the ions in the presence of the inhibitors can cause the site to lose one of the Mg^2+^, assuming that the PFV goes through a similar change in the presence of the equivalent mutation. Hare et al. also suggest that the lack of one of the ions in the crystals was likely caused by many subtle changes rather than by one major one. It is possible, however, that the addition of histidine in position 148 along with the mutation N155H destabilizes the coordination site beyond the minimum configuration required for catalysis.

### vDNA Dynamics

**FIGURE 7:**
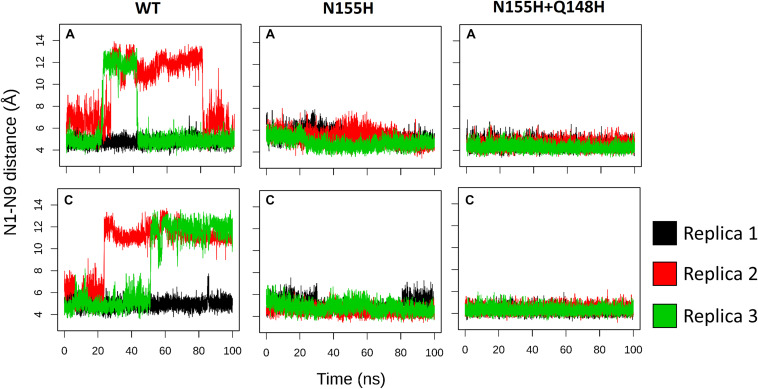
N1–N9 distance in each of the vDNA chains. The graphs show the variation of the N1-N9 distance in the vDNA chains next to chain A and chain C in each of the proteins. Each color depicts one of the replicas; replica 1, 2, and 3 are, respectively, shown in black, red, and green.

INSTIs are known for binding through an induced-fit mechanism, where the terminal nucleotide of the vDNA chain in the active site must rotate away from its position to enable binding (Hare et al., 2010b). In the crystal structure of the IN of the PFV in complex with RAL (Hare et al., 2010b), it is possible to see a difference in the terminal nucleotide position when compared with our model, showing an opening of the terminal adenine when the inhibitor is bond. Given that the rotation can occur by contributions of any of the dihedral angles along the DNA backbone to probe the rotation of the terminal adenine of the vDNA, we calculated the distance between the first Nitrogen atom of the nitrogenous base of the terminal nucleotide (N9) and the first nitrogen of the base of the previous nucleotide (N1). Thus, it was possible to assess the rotation of the terminal nucleotide about its immediate neighbor in the chain. It was observed that in two of the three MD replicas of the WT complex, the vDNA terminal close to the active site opened in both chains, moving away from the site (Figure 7). The terminal nucleotide opening can be seen in the vDNA terminal in the WT chain C in replicas 2 and 3. The N1-N9 distances in N155H show slightly higher fluctuations than in N155H+Q148, but no opening event could be seen in neither mutant.

**FIGURE 8:**
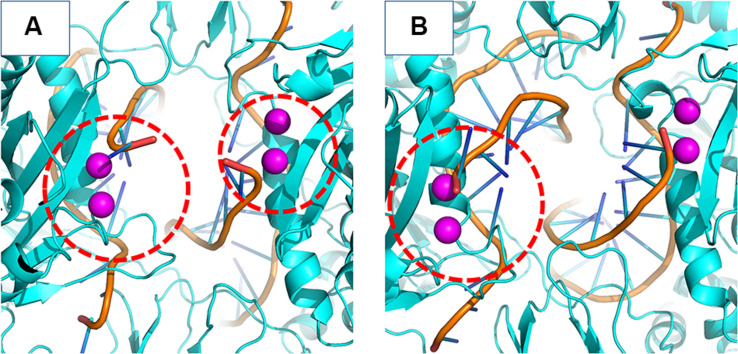
WT cSSC vDNA opening. The Mg^2+^ ions are depicted in magenta and the vDNA chain in orange. Panels **(A,B)** display two cluster centroids of the WT complex where the vDNA opening could be observed. In panel **(A)**, it is possible to see the opening in both chains, while panel **(B)** displays the opening in only one chain. The red dotted circle highlights the displaced terminal adenines.

In the crystal structure of the PFV bond to Raltegravir, the N1-N9 distance is of ≈8.6 Å, ≈9.8 Å in the SIV structure bond to Dolutegravir (Cook et al., 2020) and ≈9.4 Å in the HIV CSC bond to compound XZ419 (Passos et al., 2020), while the distance in our initial cSSC structure is of ≈5 Å. However, as seen in Figure 6, the distance explored during two of the replicas of the WT structure fluctuates around 12 Å once the opening event occurs, which is higher than what is observed in the complexes bond to INSTIs. This suggests that the presence of the inhibitor stabilizes the terminal adenine in an intermediate state since they interact with the nucleotide terminal. Two cluster centroids of the WT intasome show that the opening phenomenon exposes the ions, as can be seen in Figure 8.

This opening motion is evidence that the vDNA ends are flexible near the active site, and the presence of the INSTI is not required for the opening event to occur, but it probably stabilizes the terminal. The lack of conformational change in the terminal adenine of the N155H variant is consistent with predictions made by Hare et al. (2010b). The authors stated that the phenomenon of resistance was likely caused by the dependency of the INSTI to disrupt the interaction between the histidine in position 155 and the phosphate of the terminal nucleotide (by ≈32 KJ/mol). Indeed, in our simulations, the interaction between N155 and the terminal adenine is less favorable than the interaction between H155 and the nucleotide by ≈23 KJ/mol in the N155H and ≈25 KJ/mol in the double mutant, corroborating the previous calculations by close values. The interaction between the nucleotide and the Mgβ is also highly decreased in the mutants. These differences in the interactions may explain the vDNA opening of the WT enzyme.

## Conclusion

In the current study, our simulations suggest different Mg^2+^ coordination in the active sites of the HIV-1 IN, the N155H variant, and the double mutant N155H+Q148H. The differences in structure and energetics help explain observations from previous studies carried out with the PFV IN (Hare et al., 2010b). We believe that the different conformation of ions in the N155H variant may impact the binding mode of the INSTIs since these drugs partially rely on metal-chelating groups to properly function (Hare et al., 2010b). Possibly, other mutations that introduce long or positively charged side chains near the ions can cause resistance by a similar mechanism of Mg^2+^ ion pair disruption, for instance, in the mutants T66KI. The different coordination of the ions can also explain the deficient catalysis (Dicker et al., 2008) since not only the ions but also the catalytic residues are in different conformations. Further studies simulating the ligands in the active site of the mutants and docking calculations in the mutants could shed light into some of the details of how the differences in coordination can impair the INSTI binding.

Moreover, the dynamics of the outer chains are similar to previous MD studies that used only the IN CCD, and the so-called flexible loop and neighbor regions show different RMSF values in WT and the mutants.

Large conformational changes in the terminal nucleotide near one of the chains of the WT IN suggests that its vDNA terminal may be flexible, while no large conformational changes were observed in the terminal nucleotide of the N155H variant and the double mutant. Moreover, H155 seems to have more favorable interactions with the terminal adenine than N155. This is evidence for the hypothesis that claims N155H has stronger vDNA-IN interactions (Hare et al., 2010b). We believe that the knowledge regarding the atomic coordinates of the binding sites of resistant variants of the IN is of great value for understanding the resistance mechanisms and may help enhance the currently used drugs.

## Data Availability Statement

The datasets generated for this study are available on request to the corresponding author.

## Author Contributions

LM and AG were responsible for designing the research and writing the manuscript. LM carried out the modeling, simulation, and analysis steps. All authors contributed to the article and approved the submitted version.

## Conflict of Interest

The authors declare that the research was conducted in the absence of any commercial or financial relationships that could be construed as a potential conflict of interest.

## Supplementary Material

FIGURE S1 Final models of WT, N155H, and N155H+Q148H IN cSSC. The top and front view of all complexes detail the spatial organization of the IN monomers. The inner chains (A and C) and outer chains (B and D) are identified by color; the vDNA strands are located in the center of the complex, colored in orange.

FIGURE S2 Comparison between model and published structures. Our model is shown in green and the experimentally determined structures are shown in blue, the ions in our model are shown in magenta, and the ions from the experimentally determined structures are shown in yellow. Panel **(A)** depicts the comparison between our model and the SIV structure by Cook et al. (2020), and panel **(B)** shows the comparison between our model and the structure by Passos et al. (2020). It is possible to see an almost perfect superimposition between the Mg ions and many regions of the inner chain. The side chains of the catalytic residues (64, 116, and 152) and residues 148 and 155 are also shown, highlighting a significant superposition of the atoms of the residues.
