# Supplementary figures and images for: Evidence for Disruption of Mg2+ Pair as a Resistance Mechanism Against HIV-1 Integrase Strand Transfer Inhibitors
Source: Front Mol Biosci. 2020 Aug 20;7:170. doi: 10.3389/fmolb.2020.00170 (PMC7468422; doi:10.3389/fmolb.2020.00170)

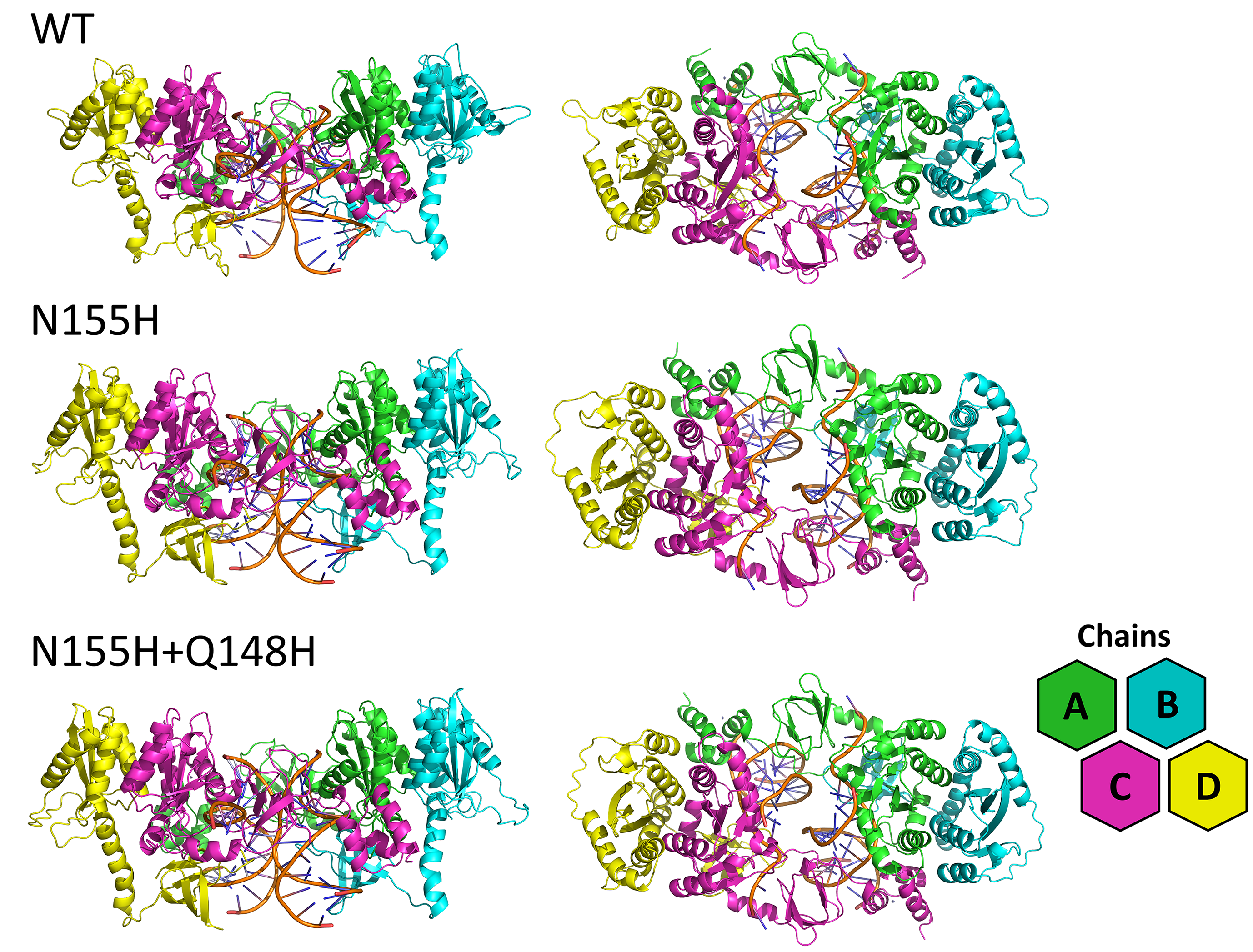

Supplement: FIGURE S1 — Final models of WT, N155H, and N155H+Q148H IN cSSC. The top and front view of all complexes detail the spatial organization of the IN monomers. The inner chains (A and C) and outer chains (B and D) are identified by color; the vDNA strands are located in the center of the complex, colored in orange. [file Image_1.TIF]

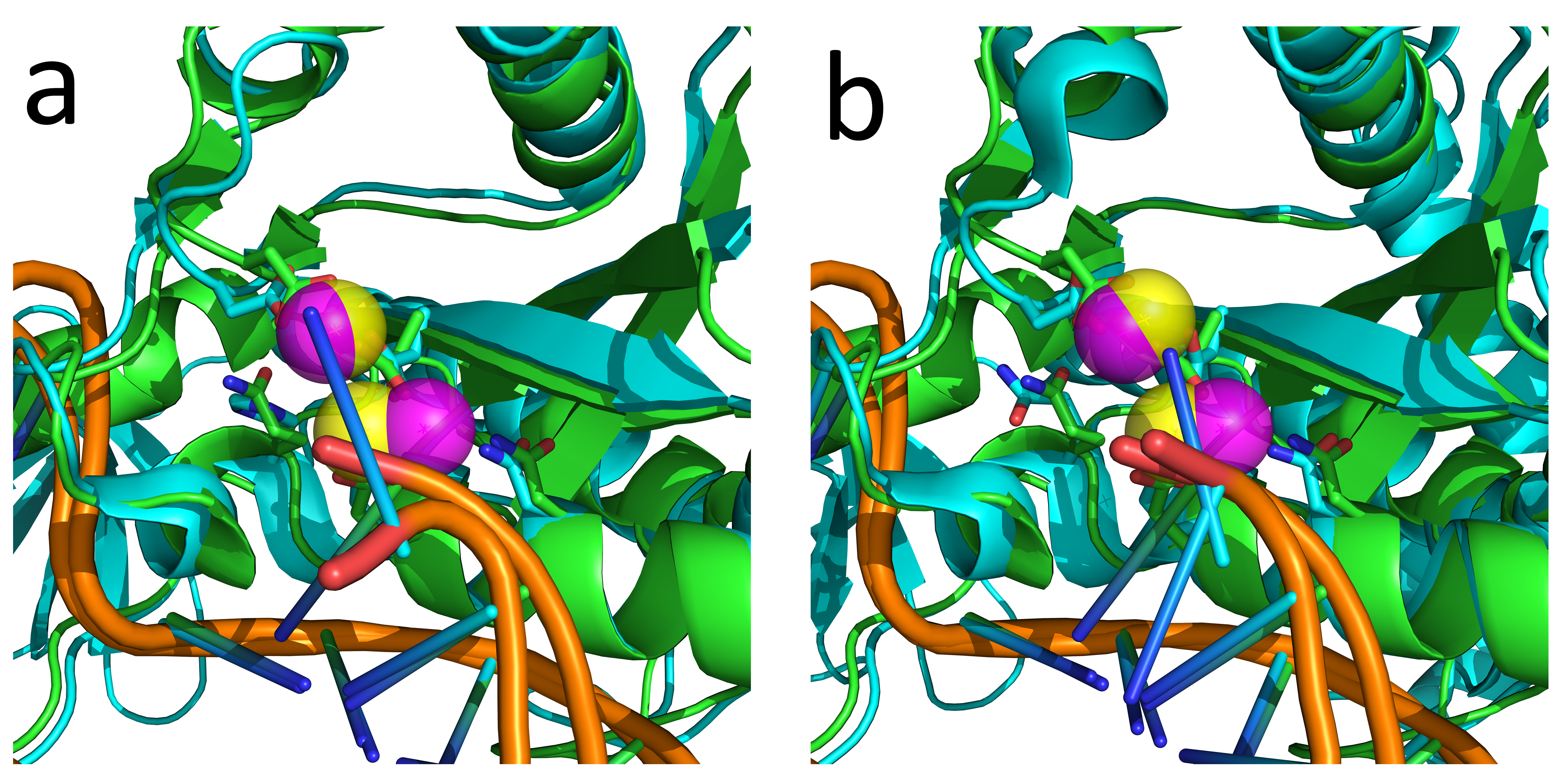

Supplement: FIGURE S2 — Comparison between model and published structures. Our model is shown in green and the experimentally determined structures are shown in blue, the ions in our model are shown in magenta, and the ions from the experimentally determined structures are shown in yellow. Panel (A) depicts the comparison between our model and the SIV structure by Cook et al. (2020), and panel (B) shows the comparison between our model and the structure by Passos et al. (2020). It is possible to see an almost perfect superimposition between the Mg ions and many regions of the inner chain. The side chains of the catalytic residues (64, 116, and 152) and residues 148 and 155 are also shown, highlighting a significant superposition of the atoms of the residues. [file Image_2.TIF]
